# Genetic structure along an elevational gradient in Hawaiian honeycreepers reveals contrasting evolutionary responses to avian malaria

**DOI:** 10.1186/1471-2148-8-315

**Published:** 2008-11-14

**Authors:** Lori S Eggert, Lauren A Terwilliger, Bethany L Woodworth, Patrick J Hart, Danielle Palmer, Robert C Fleischer

**Affiliations:** 1 Center for Conservation and Evolutionary Genetics, National Zoological Park and National Museum of Natural History, Smithsonian Institution, 3001 Connecticut Ave NW, Washington, DC 20008 USA; 2 Biological Sciences, University of Missouri, 226 Tucker Hall, Columbia, MO 65211 USA; 3 Pacific Island Ecosystems Research Center, US Geological Survey, Kilauea Field Station, PO Box 44, Hawaii National Park, HI 96718 USA; 4 25 Ocean View Ave., South Portland, ME 04106 USA; 5 Department of Biology, University of Hawaii, Hilo, HI 96720 USA

## Abstract

**Background:**

The Hawaiian honeycreepers (Drepanidinae) are one of the best-known examples of an adaptive radiation, but their persistence today is threatened by the introduction of exotic pathogens and their vector, the mosquito *Culex quinquefasciatus*. Historically, species such as the amakihi (*Hemignathus virens*), the apapane (*Himatione sanguinea*), and the iiwi (*Vestiaria coccinea*) were found from the coastal lowlands to the high elevation forests, but by the late 1800's they had become extremely rare in habitats below 900 m. Recently, however, populations of amakihi and apapane have been observed in low elevation habitats. We used twelve polymorphic microsatellite loci to investigate patterns of genetic structure, and to infer responses of these species to introduced avian malaria along an elevational gradient on the eastern flanks of Mauna Loa and Kilauea volcanoes on the island of Hawaii.

**Results:**

Our results indicate that amakihi have genetically distinct, spatially structured populations that correspond with altitude. We detected very few apapane and no iiwi in low-elevation habitats, and genetic results reveal only minimal differentiation between populations at different altitudes in either of these species.

**Conclusion:**

Our results suggest that amakihi populations in low elevation habitats have not been recolonized by individuals from mid or high elevation refuges. After generations of strong selection for pathogen resistance, these populations have rebounded and amakihi have become common in regions in which they were previously rare or absent.

## Background

The Hawaiian honeycreepers (Drepanidinae) are one of the best-known examples of an adaptive radiation. More than 50 species and subspecies are believed to have been derived from a finch-billed ancestor that colonized the islands approximately 3.5–5 million years ago [1-3]. Historically, species such as the amakihi (*Hemignathus virens*), the apapane (*Himatione sanguinea*), and the iiwi (*Vestiaria coccinea*) were found from the coastal lowlands to the high elevation forests, but by the late 1800's they had become extremely rare in habitats below 900 m in elevation [4].

While their disappearance coincided with the introduction of cattle, sheep, and goats, as well as an increase in damage to their habitat from fires and logging, these species also disappeared from low-elevation regions that appeared to be relatively undisturbed [5]. In 1968, Warner [4] proposed that the declines were the result of introduced diseases, made possible by the introduction in 1826 of the mosquito *Culex quinquefasciatus*, the primary vector of avian malaria [6]. In a series of laboratory and field experiments, van Riper *et al*. [7] established the importance of avian malaria as a limiting factor in both the abundance and distribution of native honeycreepers. Their work explained the absence of these species from low elevation habitats, where warm temperatures allow *C. quinquefasciatus *to breed year-round. Furthermore, they found that the highest malaria parasitemia levels occurred in mid-elevation habitats (900 – 1500 m), where there was overlap between native bird and mosquito breeding areas. At elevations above 1500 m, where mosquitoes were in very low densities, they observed low prevalence levels.

Malarial challenge experiments have shown that mortality rates for Hawaiian honeycreepers are remarkably high. After a single infective mosquito bite, mortality rates of high-elevation amakihi and apapane are approximately 65% [8,9]. Mortality in iiwi is higher, at approximately 90% [10]. Amakihi that survive the initial acute infection develop chronic infections and in most cases are immune to reinfection with the same isolate of the parasite [11].

This acquired immunity may provide a partial explanation for the presence today of low elevation populations of amakihi on both Oahu and Hawaii, and apapane on Hawaii [12-15]. Woodworth *et al*. [14] found that amakihi were more abundant at three low elevation forests on the southeast corner of Hawaii than at comparable high elevation forests, and that they represented 24.5% to 51.9% of the avian community. Up to 83% of the individuals in these breeding communities tested positive for avian malaria, with most experiencing low-level chronic infections. In the Puna District of Hawaii, Spiegel *et al*. [15] found that apapane were present in just under 10% of the stations they surveyed. Comparisons with studies from the 1990s suggest that amakihi, and possibly apapane, populations are expanding into areas from which they had previously been excluded by continual exposure to avian malaria [14].

If honeycreepers were largely excluded from low-elevation habitats a century ago, how can we explain the presence of large breeding populations there today? Is it possible that populations at low altitude result from recolonization of these areas by a few resistant individuals from mid or high elevation refuges? Amakihi are relatively sedentary, territorial birds that do not undergo large-scale altitudinal movements [16]. Re-colonization from high elevation habitats would most likely have taken place through dispersal in a stepping-stone fashion from high to low elevation. Alternatively, low elevation populations may have resulted from the expansion of small, isolated pockets of birds that underwent as many as 100 years of natural selection by avian malaria and other introduced mosquito-vectored pathogens. In a study using mitochondrial DNA and nuclear introns, Foster *et al*. [17] found low levels of genetic differentiation between amakihi populations within elevations, but significant differences between elevations. They found no evidence of genetic population structure in apapane or iiwi. Their results favor the hypothesis that low elevation populations of amakihi survived the introduction of avian malaria and its vector, either in multiple remnant populations or in a large and diverse population, and have since recolonized areas of their historical range.

In this study, we used nuclear microsatellite loci to further test these hypotheses in amakihi, apapane, and iiwi along an altitudinal gradient on the eastern flanks of Mauna Loa and Kilauea volcanoes on the island of Hawaii. Our results indicate that amakihi have spatially structured populations that correspond with altitude. Populations at low elevation exhibit levels of allelic diversity and heterozygosity that do not differ significantly from those of high elevation populations. These results suggest that low elevation populations are not the result of recolonization from high elevation refuges, but represent in situ host-pathogen co-evolution.

## Results

**Figure 1:**
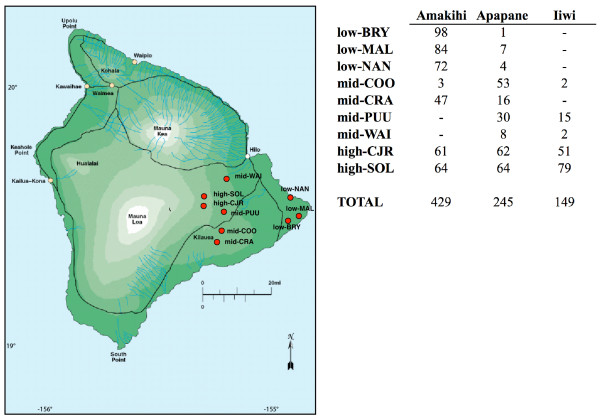
**Honeycreeper samples included in this study**. Samples were collected at nine sampling locations on the eastern slopes of the Mauna Loa and Kilauea volcanoes of the island of Hawaii. Low elevation (< 300 m above sea level) sites include Bryson's Cinder Cone (low-BRY), Malama Ki Forest Reserve (low-MAL), and Nanawale Forest Reserve (low-NAN). Mid elevation sites (between 1,000 and 1,300 m above sea level) include Cooper's (mid-COO), Crater Rim (mid-CRA), Pu'u Unit (mid-PUU), and Waiakea Forest Preserve (mid-WAI). High elevation sites (more than 1,650 m above sea level) include C. J. Ralph (high-CJR) and Solomon's (high-SOL). The numbers of samples of each species collected at each site are shown.

A total of 817 birds were genotyped for this study (Figure 1). Amakihi were found to be relatively common in low elevation sites, unlike apapane, which were rarely found there, and iiwi, which were not found at any of the low elevation sites. Amakihi were found at only two of the four mid-elevation sites, iiwi were found at three, while apapane were found at all four mid-elevation sites. All three species were found at both high-elevation sites.

### Amakihi (*Hemignathus virens*)

Levels of allelic diversity and heterozygosity were high at all sites for amakihi (Table 1). Although the sample size was very small for mid-COO, those samples were not combined with samples from the other mid-elevation site, mid-CRA, as the genetic distance between the two was surprisingly high (Table 2). Although we found deviations from expectations under HWE at several loci, there was no consistent pattern (Table 1). The possible presence of null alleles was detected at several loci, but frequencies were low in all but one case (mid-CRA, HVIR107). Due to insufficient sample size, the test for null alleles could not be done for mid-COO. There were no consistent patterns of genotypic disequilibrium between loci found in multiple populations. Values of allelic richness and private allelic richness (for all populations except COO, where sample sizes were insufficient), adjusted to a sample size of 47 (94 genes) in HP-Rare 1.0, did not differ between populations (Table 3, AMOVA, overall allelic richness p = 0.798, private allelic richness p = 0.447). Values of F_st _and R_st _(Table 2) indicate that there is significant differentiation among amakihi populations at low elevation and between these populations and all other populations except mid-COO. The population at mid-COO only differed significantly from the other mid-elevation population at mid-CRA. The mid-CRA population, however, was found to differ significantly from all other populations, with the highest levels of differentiation found between mid-CRA and low elevation populations. The two high elevation populations did not differ significantly from each other. Neither test in BOTTLENECK (SMM or TPM) detected evidence of a genetic bottleneck in any population. We found no significant correlation between genetic and geographic distance (1000 permutations in ISOLDE, Spearman Rank Coefficient p corr > obs corr = 0.316)

**Table 1:** Amakihi allelic diversity (A), expected (H_E_) and observed (H_O_) heterozygosity, and estimated frequencies of null alleles (N).

	low-BRY (n = 98)				low-MAL (n = 84)				low-NAN (n = 72)			
Locus	A	H_O_	H_E_	N	A	H_O_	H_E_	N	A	H_O_	H_E_	N
3A2C	16	0.908	0.823	-	18	0.916	0.878	-	15	0.944	0.868	-
11B1C	15	0.907	0.873	-	11	0.793	0.838	-	15	0.819	0.840	-
5A5A	15	0.827	0.897	-	16	0.831	0.865	-	15	0.889	0.891	-
4A4E	19	0.755	0.895	0.077	15	***0.627***	0.844	0.121	15	***0.597***	0.824	0.134
11B4E	10	0.763	0.737	-	12	0.643	0.658	-	10	0.789	0.730	-
12B5E	17	***0.745***	0.890	0.093	17	0.807	0.896	-	14	0.792	0.866	-
5A1B	17	***0.707***	0.897	0.111	17	0.889	0.883	-	16	***0.721***	0.878	0.088
Hvir65	14	0.845	0.889	-	14	0.780	0.887	0.076	12	0.833	0.867	-
Hvir66	20	0.857	0.875	-	21	***0.845***	0.924	0.045	18	0.806	0.884	-
Hvir94	11	0.755	0.821	-	10	0.747	0.778	-	12	0.764	0.780	-
Hvir107	9	0.680	0.818	0.084	10	0.667	0.815	0.095	8	0.653	0.790	0.069
Hvir62	5	0.592	0.623	-	6	0.646	0.616	-	5	0.500	0.564	-
*Avg*	*14.0*	*0.778*	*0.836*		*13.9*	*0.766*	*0.824*		*12.9*	*0.759*	*0.815*	
												
	**mid-COO (n = 3)**				**mid-CRA (n = 47)**							
**Locus**	**A**	**H_O_**	**H_E_**	**N**	**A**	**H_O_**	**H_E_**	**N**				
				
3A2C	5	1.000	0.855	n/a	14	***0.609***	0.807	0.116				
11B1C	7	1.000	0.963	n/a	15	1.000	0.911	-				
5A5A	3	0.750	0.605	n/a	12	***0.622***	0.857	0.137				
4A4E	5	0.500	0.785	n/a	13	0.683	0.863	0.103				
11B4E	2	0.500	0.428	n/a	7	0.574	0.696	0.096				
12B5E	5	1.000	0.858	n/a	14	***0.532***	0.791	0.169				
5A1B	6	1.000	0.928	n/a	13	0.739	.0858	0.068				
Hvir65	7	0.750	0.963	n/a	12	0.778	0.801	-				
Hvir66	4	0.750	0.785	n/a	15	***0.800***	0.899	-				
Hvir94	3	1.000	0.713	n/a	8	0.681	0.740	-				
Hvir107	4	0.750	0.820	n/a	7	***0.214***	0.799	0.347				
Hvir62	2	0.250	0.250	n/a	5	0.696	0.640	-				
*Avg*	*4.4*	*0.771*	*0.746*		*11.3*	*0.661*	*0.805*					
												
	**high-CJR (n = 61)**				**high-SOL (n = 64)**							
**Locus**	**A**	**H_O_**	**H_E_**	**N**	**A**	**H_O_**	**H_E_**	**N**				
				
3A2C	20	0.850	0.876	-	15	0.906	0.854	-				
11B1C	15	0.950	0.914	-	17	0.891	0.907	-				
5A5A	17	***0.689***	0.862	0.099	13	***0.578***	0.860	0.157				
4A4E	16	0.820	0.892	-	18	0.710	0.890	0.101				
11B4E	9	0.729	0.710	-	10	0.790	0.771	-				
12B5E	16	0.721	0.843	0.068	18	0.734	0.883	0.080				
5A1B	14	0.705	0.829	0.075	17	0.844	0.900	-				
Hvir65	14	0.820	0.900	-	18	0.906	0.922	-				
Hvir66	17	0.918	0.904	-	15	0.781	0.891	0.055				
Hvir94	9	0.689	0.814	0.074	9	0.828	0.798	-				
Hvir107	9	0.852	0.823	-	10	0.714	0.846	0.075				
Hvir62	6	0.590	0.659	-	6	0.625	0.673	-				
*Avg*	*14.0*	*0.778*	*0.836*		*14.0*	*0.778*	*0.836*					

Values that deviate significantly from expectations under Hardy Weinberg equilibrium are designated in bold italics.

**Table 2:** Genetic distances (F_st _above diagonal, R_st _below diagonal) between honeycreeper populations

A. Amakihi							
	BRY	MAL	NAN	COO	CRA	CJR	SOL
low-BRY	-	***0.0090***	***0.0082***	***0.0355***	***0.0368***	***0.0236***	***0.0201***
low-MAL	***0.0075***	-	***0.0109***	0.0273	***0.0335***	***0.0214***	***0.0190***
low-NAN	***0.0159***	***0.0168***	-	***0.0365***	***0.0388***	***0.0215***	***0.0214***
mid-COO	-0.0445	-0.0414	0.0100	-	0.0437	0.0229	***0.0332***
mid-CRA	***0.0683***	***0.0483***	***0.0789***	***0.0365***	-	***0.0322***	***0.0303***
high-CJR	***0.0227***	***0.0134***	***0.0219***	-0.0347	***0.0395***	-	0.0082
high-SOL	***0.0258***	***0.0124***	***0.0286***	-0.0432	***0.0244***	0.0034	-
							
**B. Apapane**							
	LOW	COO	CRA	PUU	WAI	CJR	SOL

low- BRY/MAL/NAN	-	***0.0298***	***0.0289***	***0.0311***	***0.0327***	***0.0352***	***0.0259***
mid-COO	***0.0866***	-	0.0047	-0.0014	***0.0165***	***0.0067***	***0.0053***
mid-CRA	***0.0518***	-0.0059	-	0.0627	-0.0040	0.0038	0.0034
mid-PUU	***0.0626***	-0.0006	-0.0201	-	0.0098	0.0019	0.0050
mid-WAI	***0.0977***	***0.1133***	0.0457	0.0797	-	0.0073	0.0019
high-CJR	***0.0596***	***0.0192***	-0.0149	0.0006	0.0401	-	0.0073
high-SOL	***0.0416***	***0.0450***	0.0076	0.0280	0.0306	***0.0118***	-
							
**C. Iiwi**							
	MID	CJR	SOL				

Mid- COO/PUU/WAI	-	***0.0082***	-0.0011				
CJR	***0.0232***	-	0.0073				
SOL	0.0147	0.0000	-				

Values shown in bold italics were found to be significantly different from zero (p < 0.05) using permutation tests (1023 permutations) in ARLEQUIN.

**Table 3:** Adjusted values of allelic richness A and private allelic richness A_P _for amakihi

	low-BRY		low-MAL		Low-NAN		mid-CRA		high-CJR		high-SOL	
LOCUS	A	A_P_	A	A_P_	A	A_P_	A	A_P_	A	A_P_	A	A_P_
**3A2C**	13.4	0.0	15.3	2.0	14.4	0.0	13.0	1.0	17.6	4.6	14.5	1.1
**11B1C**	13.2	0.0	10.8	0.0	13.9	1.2	15.0	0.0	14.9	0.1	16.1	1.2
**5A5A**	13.7	0.2	13.6	0.1	12.7	0.8	12.0	0.5	15.7	2.3	12.4	0.9
**4A4E**	14.6	0.9	13.6	0.3	12.9	0.9	13.0	0.1	15.4	0.0	16.7	0.9
**11B4E**	9.0	0.5	9.9	1.6	9.4	0.6	7.0	0.0	8.7	0.0	9.7	0.7
**12B5E**	14.7	0.7	14.8	1.1	12.7	0.0	13.9	0.1	14.6	0.7	16.8	1.7
**5A1B**	15.3	0.0	14.9	0.0	14.8	0.7	13.0	0.0	12.9	0.1	15.6	0.1
**HVIR65**	13.0	0.5	12.9	0.8	11.5	0.0	12.0	0.0	13.6	0.0	17.2	1.1
**HVIR66**	16.8	0.6	18.5	0.5	15.9	0.0	15.0	0.3	16.4	0.3	14.7	0.0
**HVIR94**	10.4	0.3	9.4	0.1	10.9	1.4	8.0	0.0	8.7	0.1	8.6	0.3
**HVIR107**	8.7	0.0	8.8	0.6	7.5	0.2	7.0	0.3	8.7	0.0	9.1	0.3
**HVIR62**	4.9	0.0	5.1	0.0	4.6	0.0	5.0	0.0	5.9	0.0	5.9	0.0
												
**AVERAGE**	12.3	0.3	12.3	0.6	11.8	0.5	11.2	0.2	12.8	0.7	13.1	0.7

Values were computed using rarefaction in HP-RARE 1.0; due to small sample size, the population at mid-COO was not included in this analysis.

**Figure 2:**
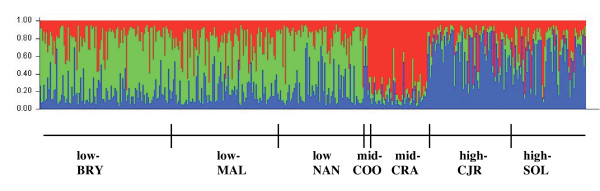
**Results of the analysis of Amakihi microsatellite genotypes in STRUCTURE**. Three genetic groupings were detected, indicating population division along an altitudinal gradient.

Analysis in STRUCTURE detected three genetic clusters of amakihi (Table 4), indicating population subdivision along an altitudinal gradient (Figure 2). The first cluster includes individuals from all populations at low altitude. Birds captured at mid-CRA were assigned to the second cluster. The third cluster is made up of birds captured at high altitude. The mid-COO birds were found to be more similar to high elevation birds than to low elevation birds or birds at mid-CRA.

**Table 4:** Results of analysis of the data in STRUCTURE 2.2

K	Avg L(K)	Δ**K**
**Amakihi**		
1	-22805.2	n/a
2	-22791.6	2.4
3	-22464.0	22.8
4	-22961.6	3.0
5	-22650.8	3.2
6	-23277.5	2.9
7	-23683.3	1.1
**Apapane**		
1	-11541.2	n/a
2	-12256.4	1.8
3	-12323.1	2.5
4	-12302.1	2.0
5	-12265.1	2.3
6	-12265.8	2.9
7	-12206.6	1.5
**Iiwi**		
1	-6945.9	n/a
2	-7075.6	7.4
3	-8071.8	3.9
4	-7620.8	2.5
5	-7839.7	1.8
6	-7820.1	5.2
7	-9317.7	2.3

Average values of the log likelihood of the data [Ln P(D)] for K = 1–7, and values of ΔK for each.

### Apapane (*Himatione sanguinea*)

Despite intensive sampling efforts, few apapane were sampled at low elevation. The twelve samples collected in the low elevation sites were pooled for the analyses, after preliminary data revealed no differences in allele frequencies (data not shown). Levels of allelic diversity and heterozygosity were high in apapane (Table 5). Although there were deviations from expectations under HWE at several loci, there was no consistent pattern (Table 5). The possible presence of null alleles was detected at several loci, but frequencies were low in all but one case (low, HVIR65). Genotypic disequilibrium was not found between pairs of loci in multiple populations.

**Table 5:** Apapane allelic diversity (A), expected (H_E_) and observed (H_O_) heterozygosity, and estimated frequencies of null alleles (N).

low-BRY, MAL, NAN (n = 12)				Mid-COO (n = 53)				Mid-CRA (n = 16)				
Locus	A	H_O_	H_E_	N	A	H_O_	H_E_	N	A	H_O_	H_E_	N
3A2C	10	0.750	0.873	-	14	0.717	0.865	0.083	13	***0.563***	0.899	0.178
11B1C	12	1.000	0.931	-	15	0.906	0.872	-	11	0.875	0.887	-
5A5A	8	0.750	0.789	-	17	0.906	0.882	-	14	0.938	0.935	-
4A4E	5	0.600	0.663	-	13	***0.600***	0.829	0.130	7	0.400	0.731	0.193
11B4E	5	0.750	0.710	-	6	0.698	0.733	-	6	0.625	0.752	-
12B5E	7	0.917	0.869	-	21	0.887	0.918	-	12	0.875	0.908	-
5A1B	7	0.833	0.837	-	12	0.736	0.879	0.075	11	0.688	0.905	0.106
Hvir65	3	0.333	0.625	0.204	9	0.679	0.689	-	5	0.750	0.653	-
Hvir66	11	1.000	0.920	-	14	0.906	0.924	-	12	0.938	0.926	-
Hvir94	2	0.333	0.289	-	3	0.528	0.513	-	2	0.375	.0508	-
Hvir107	6	0.917	0.840	-	14	0.774	0.889	-	8	0.625	0.873	0.128
Hvir62	3	0.500	0.423	-	4	0.377	.0342	-	3	0.188	0.179	-
*Avg*	*6.6*	*0.724*	*0.731*		*11.8*	*0.726*	*0.778*		*8.7*	*0.653*	*0.763*	
												
	**mid-PUU (n = 30)**				**mid-WAI (n = 8)**							
**Locus**	**A**	**H_O_**	**H_E_**	**N**	**A**	**H_O_**	**H_E_**	**N**				
				
3A2C	12	***0.567***	0.866	0.166	7	0.750	0.850	-				
11B1C	14	0.867	0.886	-	7	0.875	0.841	-				
5A5A	13	0.833	0.884	-	7	0.875	0.850	-				
4A4E	12	0.607	0.844	0.137	6	0.625	0.783	-				
11B4E	6	0.867	0.760	-	5	1.000	0.813	-				
12B5E	17	0.862	0.913	-	10	1.000	0.934	-				
5A1B	13	0.700	0.906	0.106	6	0.750	0.850	-				
Hvir65	5	0.700	0.691	-	5	0.875	0.733	-				
Hvir66	13	0.967	0.919	-	10	1.000	0.925	-				
Hvir94	2	0.467	0.508	-	3	0.500	0.491	-				
Hvir107	14	0.833	0.890	-	6	0.714	0.857	-				
Hvir62	5	0.267	0.299	-	2	0.250	0.234	-				
*Avg*	*10.5*	*0.711*	*0.781*		*8.7*	*0.653*	*0.763*					
												
	**high-CJR (n = 62)**				**high-SOL (n = 64)**							
**Locus**	**A**	**H_O_**	**H_E_**	**N**	**A**	**H_O_**	**H_E_**	**N**				
				
3A2C	15	0.738	0.905	0.086	14	0.776	0.896	0.062				
11B1C	13	0.839	0.886	-	13	0.862	0.893	-				
5A5A	17	0.855	0.893	-	16	0.917	0.880	-				
4A4E	15	0.672	0.824	0.083	13	***0.576***	0.846	0.150				
11B4E	6	0.705	0.766	-	6	0.695	0.723	-				
12B5E	20	0.968	0.907	-	22	0.931	0.901	-				
5A1B	15	0.836	0.897	-	15	0.763	0.891	0.067				
Hvir65	9	0.726	0.780	-	9	0.576	0.673	-				
Hvir66	14	0.919	0.907	-	16	0.883	0.931	-				
Hvir94	3	0.410	0.511	0.092	6	0.356	0.511	0.146				
Hvir107	13	0.806	0.877	-	12	0.810	0.865	-				
Hvir62	4	0.194	0.222	-	5	0.513	0.362	-				
*Avg*	*12.0*	*0.722*	*0.781*		*12.3*	*0.722*	*0.781*					

Values that deviate significantly from expectations under Hardy Weinberg equilibrium are designated in bold italics.

Values of F_st _and R_st _(Table 2) indicate that there is significant differentiation between apapane populations at low elevation and all other populations at mid and high elevations. The mid-elevation population at mid-COO differed significantly from the mid-elevation population at mid-WAI, as well as from the high elevation populations. The two high elevation populations did not differ significantly from each other. We found no significant correlation between genetic and geographic distance (1000 permutations in ISOLDE, Spearman Rank Coefficient p corr > obs corr = 0.150). Analysis in STRUCTURE found only a single genetic group of apapane (K = 1, Table 4).

### Iiwi (*Vestiaria coccinea*)

Although they appear to be slightly lower, levels of allelic diversity were not significantly different in iiwi than in amakihi or apapane (ANOVA, p = 0.357), and heterozygosity values were comparable to the other species (Table 6). There were no significant deviations from heterozygosity values expected under HWE, and MICRO-CHECKER did not detect null alleles at any locus. No consistent patterns of genotypic disequilibrium were found between loci.

**Table 6:** Iiwi allelic diversity (A), expected (H_E_) and observed (H_O_) heterozygosity, and estimated frequencies of null alleles (N).

	mid-COO, PUU, WAI (n = 19)				high-CJR (n = 51)				mid-SOL (n = 79)			
Locus	A	H_O_	H_E_	N	A	H_O_	H_E_	N	A	H_O_	H_E_	N
3A2C	5	0.684	0.631	-	8	0.714	0.667	-	10	0.513	0.616	-
11B1C	11	0.789	0.873	-	15	0.780	0.825	-	20	0.838	0.893	-
5A5A	7	0.789	0.777	-	8	0.843	0.834	-	12	0.813	0.817	-
4A4E	10	0.882	0.855	-	14	0.854	0.873	-	15	0.797	0.866	-
11B4E	6	0.579	0.622	-	7	0.784	0.725	-	9	0.563	0.595	-
12B5E	8	0.684	0.799	-	14	0.686	0.794	-	13	0.700	0.792	-
5A1B	7	0.647	0.774	-	11	0.804	0.852	-	11	0.910	0.868	-
Hvir65	5	0.778	0.757	-	10	0.824	0.819	-	11	0.797	0.820	-
Hvir66	6	0.895	0.782	-	7	0.820	0.820	-	11	0.859	0.839	-
Hvir94	15	1.000	0.912	-	17	0.902	0.913	-	19	0.911	0.916	-
Hvir107	11	1.000	.0916	-	15	0.961	0.913	-	17	0.897	0.906	-
Hvir62	4	0.588	0.511	-	6	0.510	0.597	-	5	0.557	0.522	-
*Avg*	*7.9*	*0.776*	*0.767*		*11.0*	*0.790*	*0.803*		*12.8*	*0.763*	*0.787*	

Values that deviate significantly from expectations under Hardy Weinberg equilibrium are designated in bold italics.

Values of F_st _and R_st _(Table 2) indicate that there is slight but significant differentiation between iiwi at mid elevation and those at high-CJR. The two high elevation populations did not differ significantly from each other. Analysis in STRUCTURE found only a single genetic group of Iiwi (K = 1, Table 4).

## Discussion

Until relatively recently, studies by highly qualified scientists [4,16,18-20] found that honeycreeper populations were confined to regions above 600 m. Thus, it was both surprising and exciting that Woodworth *et al*. [14] and Spiegel *et al*. [15] detected large breeding populations of amakihi and smaller, more patchily distributed groups of apapane in low elevation habitats.

If honeycreepers were largely excluded from low-elevation habitats a century ago, how can we explain the presence of large breeding populations of amakihi there today? One hypothesis is that birds from high elevation that were genetically resistant to avian malaria have re-colonized low elevation habitats. Because amakihi are relatively sedentary, territorial birds that do not undergo large-scale altitudinal movements [16], re-colonization from high elevation habitats would most likely have taken place through dispersal in a stepping-stone fashion from high to low elevation. From malarial challenge experiments [8], we know that today only approximately 35% of amakihi from high elevation populations survive infection under optimal conditions in the aviary. Thus, we would expect strong selection to continually reduce the numbers of dispersers once they enter regions where the parasite and its vector breed. This protracted bottleneck would result in low elevation populations that are genetically similar to high elevation populations, but that have reduced allelic diversity and possibly an excess of heterozygosity, since the rare alleles that are lost affect the predicted heterozygosity values but not the observed values [21].

Our results do not support this hypothesis. Low elevation populations of amakihi were found to be genetically distinct from populations at mid and high elevations, and the levels of both allelic diversity and private alleles did not differ between low, mid, and high elevations. We found no evidence for isolation by distance, nor did we detect an excess of heterozygosity in either mid or low elevation populations. Instead, we found that the mid elevation population at mid-CRA had a number of loci that did not conform to HWE expectations due to reduced heterozygosity.

An alternate (but not exclusive) hypothesis is that low elevation amakihi populations were founded by individuals that survived the introduction of pathogens approximately 100 years ago. Our results are more consistent with this hypothesis. At that time, low elevation populations were becoming fragmented as a result of logging and habitat conversion. Losses of large numbers of individuals to disease would likely have resulted in small, isolated pockets of resistant birds. These small populations would have been at extreme risk for extinction, but after a number of generations of strong selection in favor of resistant genotypes, enough offspring may have survived for population growth. Although the survivors may have shared alleles at genes that conferred resistance, the isolated groups were less likely to have shared alleles at neutral loci. Secondary contact between these isolates would explain the presence of genetically distinct populations at low elevation with levels of allelic diversity and private alleles that are comparable with populations at mid or high elevation. Such small pockets may have been much less detectable by naturalists, hence their reports of no native birds at low elevations.

Further support for this hypothesis comes from the observation that the mid elevation population of amakihi at mid-CRA differs genetically from populations at both low and high elevation. Avian malaria may have only invaded this region recently, as its mosquito vector reacted to increasing temperatures by expanding its range up-slope [22,23]. The fact that small populations of amakihi persist there suggests that there may be individuals that possess alleles for resistance, but that the frequency of those alleles has not reached a level at which a sufficient number of offspring survive to allow the populations to recover. In the absence of gene flow from resistant populations, mid-elevation populations remain small. Our observation that five of the twelve microsatellite loci deviated from HWE expectations in the mid-CRA population supports the notion that this is not a population at mutation/drift equilibrium.

While the sedentary habits of the amakihi may have contributed to their survival in low elevation habitats, the larger altitudinal movements of apapane and iiwi may increase their exposure rates to introduced disease [7,24]. Apapane at low elevation were genetically distinct from those at other elevations, though these results should be viewed with caution, as sample sizes at low elevation sites were small. It is possible that low elevation populations have been founded by a few resistant birds from higher elevation. Growth of these populations through breeding between resistant individuals may be hindered if migration levels from higher elevations are significant. Conversely, high elevation populations may not be protected by distance from regions in which conditions are favorable for infection, due to movement of individuals in and out of the low and especially mid elevational habitats.

We detected slight but significant differentiation between iiwi at mid elevation and those at high-CJR. As with apapane at low elevation, these results should be viewed with caution as very few samples could be obtained at mid elevation and fewer samples could be obtained at high-CJR than at high-SOL. Previous studies using mitochondrial DNA RFLP analyses [1] and control region sequences [25] found no genetic variation in iiwi. Our finding of high allelic diversity and heterozygosity at microsatellite loci is exciting, and is similar to the finding of high diversity at MHC Class II β peptide-binding codons in this species [25]. Despite these high levels of neutral and adaptive nuclear diversity, Atkinson *et al*. [10] found that mortality after experimental infection with the avian malaria parasite is approximately 90% in iiwi. Assuming that resistance has a genetic basis, the alleles that confer resistance appear to be rare. If the prevalence of avian malaria increases in high elevation habitats due to climate change, this species will be highly vulnerable [26]. There is some indication, however, that the few iiwi that recover from acute malarial infection can successfully breed in subsequent years [23].

## Conclusion

Our results indicate that these three species have had different responses to the introduction of exotic pathogens, as suggested by van Riper *et al*. [7]. What he and his colleagues did not envision, however, was that at low elevation amakihi may not have been eliminated, but may have survived in small, isolated populations. Our results suggest that amakihi were present at low elevation, but were rarely detected during limited surveys by highly qualified researchers. We suggest that after generations of strong selection for pathogen resistance, these populations have rebounded and amakihi have spread and become common in regions in which they were previously rare or absent. This success story provides opportunities for us to better understand the complexities of host-pathogen dynamics as well as the mechanisms of the evolution of the host-pathogen relationship. It also provides data that will help us to design long-term strategies for the conservation and restoration of honeycreeper species to their native habitats.

## Methods

Samples were collected from nine 1 km^2 ^study sites within an approximately 1,100 km^2 ^region on the eastern slopes of the Mauna Loa and Kilauea volcanoes (Figure 1). Study sites were distributed along an elevational gradient from 25–1800 m, and were stratified into three elevational classes, with two replicates at high elevation (greater than 1650 m above sea level (ASL)): C. J. Ralph (high-CJR) and Solomon's (high-SOL); four replicates at mid elevation (1000–1300 m ASL): Cooper's (mid-COO), Crater Rim (mid-CRA), Pu'u Unit (mid-PUU) and Waiakea (mid-WAI); and three at low elevation (less than 300 m ASL): Bryson's (low-BRY), Malama Ki (low-MAL) and Nanawale (low-NAN). These classes correspond to the 3 major disease "zones" identified by van Riper *et al*. [7]; where "low elevation" corresponds to low native bird abundance, high avian malaria transmission rates, and high prevalence of avian malaria; "mid elevation" corresponds to moderate native bird abundance, and seasonally high transmission and infection rates; and "high elevation" corresponds to high native bird abundance, and very low transmission and infection rates. Birds were captured in mist-nets between April 2001 and July 2003, with the majority of samples (95%) drawn from birds captured in 2002. All birds were processed according to standard mistnetting protocol and banded for individual identification. Upon capture, a 100 μl blood sample was taken by jugular venipuncture with a heparinized 28-gauge insulin syringe for malarial diagnostics and genetic analysis. Blood was spun with a portable centrifuge to separate plasma from red blood cells. Separated red blood cells were deposited into lysis buffer in individual 1.5 ml plastic tubes and subsequently frozen at -20°C.

DNA was extracted from blood samples using the DNeasy Blood and Tissue Kit (Qiagen). Samples were genotyped at twelve microsatellite loci, seven of which were developed for another Hawaiian honeycreeper, the Laysan finch (*Telespiza cantans*): 3A2C, 4A4E, 5A1B, 5A5A, 11B1C, 11B4E, 12B5E [27]. The remaining five were characterized in the amakihi: Hvir62, Hvir65, Hvir66, Hvir94, and Hvir107 [28]. All loci contain dinucleotide repeats except Hvir107, which contains a tri-nucleotide repeat region.

Genotyping was performed in 10 μl volumes containing 1 μl AmpliTaq Gold 10X DNA polymerase buffer (Applied Biosystems, Inc.), 0.5 μM fluorescently labeled forward primer, 0.5 μM unlabeled reverse primer, 2 μM each dNTP, 10 mM MgCl2, and 0.5 U AmpliTaq Gold polymerase, to which was added approximately 15 ng genomic DNA. The PCR was performed in a PTC-100 or PTC-200 thermocycler (MJ Research, Inc.) and included a 10 min preincubation at 95°C to denature the DNA and activate the polymerase, followed by 35 cycles of denaturation at 95°C for 45 sec, primer annealing at locus-specific temperatures for 35 sec, and primer extension at 72°C for 35 sec, and a final 7 min extension at 72°C. All reactions included a Laysan finch sample that was used to standardize allele sizes between runs and species, and a control reaction with no DNA to detect possible contamination of the PCR. Amplification products were analyzed in an ABI 3100 automated DNA sequencer (Applied Biosystems, Inc.) using GeneScan 3.7 (Applied Biosystems, Inc.). Fragment sizes were scored by comparison with ROX 500 size standards in Genotyper 2.5 (Applied Biosystems, Inc.).

Genotypic data were analyzed in GENEPOP 3.1b [29], where observed and expected heterozygosities were determined for all loci, exact tests were performed to detect significant deviations from expectations under Hardy-Weinberg equilibrium (HWE), linkage disequilibrium between loci was assessed, and genetic differentiation between populations (F_st_) was calculated. The program ISOLDE, also incorporated in GENEPOP, was used to assess correlations between geographic distances [ln (distance)] and genetic distances [F_st_/(1-F_st_)]. The presence and frequency of null alleles was assessed in MICRO-CHECKER[30]. We computed the genetic distances F_st _[31,32] and R_st _[33] between populations and elevations, assessed their significance levels using permutation tests, and performed analysis of molecular variance (AMOVA) tests in ARLEQUIN 2.000 [34]. Because sample sizes differed between amakihi populations, we used HP-RARE 1.0 [35] to compare overall and private allelic diversity between amakihi populations. To detect the presence of possible population bottlenecks, genotype data were analyzed in the computer program BOTTLENECK[36] using the stepwise mutation model (SMM) and two-phase model (TPM) at 95% stepwise and 70% stepwise.

To examine population structure, we used STRUCTURE Version 2.2 [37], which uses a Bayesian clustering approach and no prior information on the sampling sites of individuals. For each K (number of genetic clusters) from 1 to 7, we ran the program 3 times, using a burn-in length of 100,000 and 500,000 simulations. Using the admixture model, we estimated the proportions of their genome that individuals of mixed ancestry inherited from ancestors in each of the resulting clusters, and averaged that over individuals in each population. We used both the log likelihood of the data [Ln P(D)] values and the ΔK statistic [38] to determine the number of genetic clusters for each species.

## Authors' contributions

LSE developed 5 of the microsatellite loci used in this project, carried out the molecular genetic studies, analyzed the data, and drafted and revised the manuscript. LAT assisted LSE with the molecular genetic studies and the analysis of the results. BLW directed the sampling efforts for this study and contributed to drafting and revising the manuscript. PJH led the field team, assisted with the spatial analyses, and contributed to drafting and revising the manuscript. DP assisted with the molecular genetic studies and the analysis of the results. RCF conceived of the molecular genetics study, directed the laboratory work and data analyses, and contributed to drafting and revising the manuscript.
